# Supplementary material for: Lower Ordovician synziphosurine reveals early euchelicerate diversity and evolution
Source: Nat Commun. 2024 May 7;15:3808. doi: 10.1038/s41467-024-48013-w (PMC11076625; doi:10.1038/s41467-024-48013-w)
Supplement: Supplementary file 1 — Supplementary Information [file 41467_2024_48013_MOESM1_ESM.pdf]

Lower Ordovician synziphosurine reveals early euchelicerate diversity and evolution

Lorenzo Lustri, Pierre Gueriau, and Allison C. Daley

Supplementary Information

This Supplementary Information file includes:

- Supplementary Discussion 2
- Supplementary Notes 8
- 17 Supplementary Figures 10
- 3 Supplementary Tables 27
- Supplementary References 29

SUPPLEMENTARY DISCUSSION

Jianshania furcatus Luo *et a*l., 1999

Further insights into early euchelicerate character evolution may come from poorly-known Cambrian taxa exhibiting body plans similar to that of Offacolidae, such as *Jianshania furcatus* from the Chengjiang biota [1]. *Jianshania furcatus* seems to possess most dorsal characters of Offacolidae, with eleven trunk tergites, a fully expressed first tergite and a developed axis (a combination which is unique within euchelicerates to *Setapedites abundantis* so far), and a bifurcate telson [1]. Unfortunately, its ventral anatomy remains unknown, requiring investigation using microtomographic imaging, which has provided pivotal new information on the ventral anatomy of other Chengjiang arthropods [1-3], to decipher its relationships with Cambrian taxa and Offacolidae, and to document the evolution of some of the derived characters observed in *Habelia optata*.

Pretelsonic process

Some specimens of *Setapedites abundantis* reveal an ovoid bipartite process under the eleventh opisthosomal tergite (Figs. 2A, 2B, 3A and 3B, Supplementary Figs. 1A–F, 3E and 3F), the rare and thin preservation of which suggests that this process was not strongly sclerotized, as sclerotized structures are commonly preserved in Fezouata taxa [4]. In *Habelia optata*, an anal pouch is present on its last abdominal somite [5], in the same position as the pretelsonic process in *Setapedites abundantis*. There are multiple possible interpretations for this process based on the currently available data. A first hypothesis is that this process represents an anal pouch, similar to that in *Habelia* [5]. A second hypothesis is that the pretelsonic process present in *Setapedites abundantis* may be homologous to the posterior modified appendages characteristic of Vicissicaudata[6,7]. Such an interpretation would require the loss of appendicular derivatives in many linages independently according to our phylogenetic results and previous research [8]. A third hypothesis is that all of these appendicular derivatives (habeliid anal pouch, vicissicaudate appendicular derivatives and offacolid post ventral process) are homologues, and represent an apomorphy of a monophyletic Chelicerata+ Vicissicaudata+ Habeliida. Until further fossil evidence can be produced, we prefer to keep a neutral anatomy-focused description of this process, without over-interpretation of its putative phylogenetic significance.

The labrum in *Setapedites abundantis* and other offacolids

The oral region of *Setapedites abundantis* is preserved in a few specimens, and only two preserve anatomical information that we can use to understand the anatomy of the labrum and its position with

respect to the appendages. The structure described as a labrum in *Setapedites abundantis* is not unique among the Offacolidae. *Offacolus kingi* is described as displaying a "bulge" in the same position [13] and we regard it to be homologous to the labrum described in *Setapedites abundantis* (lb in Fig. 2A–D, I and J, Supplementary Fig. 2B and 2C). The position of the labrum in *Setapedites abundantis* is comparable with the position of this structure in *Offacolus kingi* [9] and extant Xiphosurida [10] with respect to the insertion of the chelicera. It is interesting to note that the shape of the labrum described in Offacolidae resembles that of megacheiran euarthropods such as *Leanchoilia illecebrosa* [3]. The absence of the eyes in Offacolidae prevents a comparison of the relative position of the labrum in the head structure of offacolids and megacheirans, making the establishment of their homology problematic. If future study can confirm the homology of the offacolid and megacheiran labrum, that would represent strong support for affinities of megacheirans with the Euchelicerata. Anterior to the labrum, a poorly preserved secondary structure is present (Fig. 2A–D, I and J, Supplementary Fig. 2B and 2C). This structure may be an epistome, homologous to that of extant xiphosurids. However, an alternative interpretation for this ill-defined structure may be provided by a comparison with *Offacolus kingi* and *Dibasterium durgae*. In both these other offacolids, the first post-cheliceral endopod inserts close to the chelicera. As seen in Supplementary Fig. 2B and 2C, the apparently rounded shape of the pre-labrum structure present in the counterpart of the specimen figured in Fig. 2A–D instead may be an artifact and could originate from the small gnathobase of the prosomal appendages inserted centrally into the prosoma. Also, in this case, until further specimens provide more information regarding the anatomy of the oral region, we prefer to keep a neutral approach and only state the presence of this anatomical feature without assigning a definitive interpretation to this structure associated with the labrum.

Anatomical comparison of *Setapedites abundantis* with non-offacolid synziphosurines, aglaspidids and *Saratrocerus oblitus*

Aside from Offacolidae, the ventral anatomy of *Weinbergina opitzi* Richter and Richter, 1929 [11-13] is also well known. *Weinbergina opitzi* may potentially share biramous prosomal appendages with the offacolids [14]. The termination of the post-cheliceral limbs of *Weinbergina opitzi* is not chelate, but instead, bears setae similar to the brush-like last podomere characteristic of offacolids prosomal exopods. This could imply that the appendages described so far in *Weinbergina opitzi* have an exopodial nature, and subsequently imply a loss of the endopods or that the endopods have not yet been identified in *Weinbergina opitzi*. Anatomical comparison of only the dorsal morphology has led to a poor understanding of the phylogenetic relationships of the euchelicerates informally known as 'synziphosurines'. However, for most of the taxa informally termed 'synziphosurines', this is the only

anatomical feature available because the ventral anatomy has not been recorded. If we compare the dorsal anatomy of *Setapedites abundantis* to that of non-offacolid synziphosurines, three taxa show a comparable dorsal anatomy: *Pasternakevia podolica* [15], *Cyamocephalus loganensis* [16,17] and *Bunodes lunula* [18].

Pasternakevia podolica shares with *Setapedites abundantis* a strongly developed axis and the general shape of the tergopleural spines, alongside the hypertrophic second tergite (this character is extreme in *Pasternakevia podolica* while it is less developed in *Setapedites abundantis*) but it differs in the presence of a reduced first tergite (microtergite), the absence of ring-like abdominal sclerites are devoid of pleurae and possibly the reduced number of tergites (presumably ten in *Pasternakevia podolica* while in *Setapedites abundantis* there are eleven) [19].

Cyamocephalus loganensis instead shares with *Setapedites abundantis* the prosomal morphology, with an apical spine, cardiac lobe (median ridge) and sunken region (ophthalmic ridge), as well as the developed axis and the general shape of the pleural spines. However, it differs in the presence of a reduced first tergite, the absence of ring-like abdominal sclerites devoid of pleurae and the number of tergites forming the opisthosoma.

Finally, *Bunodes lunula* shares with *Setapedites abundantis* most of its dorsal anatomy. The prosoma shows a similar lunate shape and displays a cardiac lobe (median ridge) and sunken region (ophthalmic ridge). In *Setapedites abundantis* (and *Dibasterium*) the body is divided in a pre-abdomen formed by tergites with a developed axis and tergopleural spines, followed by an abdomen made of ring-shaped sclerites devoid of pleura. However, it is differentiated by the presence of a reduced first tergite (microtergite) and the total number of tergites and a thicker, more conical telson devoid of bifurcation.

The shared anatomical features with *Setapedites abundantis* make all of these taxa candidates to be included in Offacolidae, particularly *Bunodes lunula*. New studies aiming to resolve the appendicular anatomy of those taxa may resolve their phylogenetic position. On the other hand, the comparison of those taxa with *Setapedites abundantis* shows that features such as the microtergite and opisthosoma reduced from eleven to ten segments are derived characters of *Pasternakevia, Cyamocephalus* and *Bunodes*.

It is also worth mentioning the similarity between *Setapedites abundantis* and the aglaspidids belonging to the genus *Tremaglaspis.* Aglaspidida is a poorly understood clade of Vicissicaudata, and as such is difficult to assess their relationships with other euarthropods. However, the dorsal morphology of the genus *Tremaglaspis* shows significant similarities with *Setapedites abundantis. Termaglaspis* includes two described species, *Tremaglaspis unite* Fortey and Rushton, 2003 from the lower Ordovician of Wales[20] and *Tremaglaspis vanroyi* Lerosey-Aubril *et al.,* 2013 from the middle

Cambrian of Utah (USA)[21]; a third, presently undescribed species is present in the Fezouata biota [22-24]. These species share with *Setapedites abundantis* the generally elongated body divided into a cephalon (prosoma), a trunk (opisthosoma) made of 11 tergites with a raised axial region and tapering posteriorly, and a spinose telson. Another possible shared character is the presence of a postventral plate at least in *Tremaglaspis unite*, which shares similar symmetry and position with the pretelsonic process *of Setapedites abundantis*. However, those species differ from *Setapedites abundantis* in the absence of a concave region and median ridge in the cephalon, the absence of bipartite tergites and subaxial nodes, the presence of anterior tergal processes in at least the undescribed tremaglaspidid from the Fezouata Biota [22], a less defined division of the trunk into an abdomen and post-abdomen and a wider telson. Those differences, alongside the absence of knowledge regarding *Tremaglaspis* ventral anatomy, allow only a general acknowledgment of their superficial similarity and as such underline the need for new data regarding Aglaspidida.

Finally, we consider *Sarotrocercus oblitus*, which has been recovered in some phylogenies as possible a chelicerate [8,25]. According to its most recent redescription[26], even though this taxon resembles the Offacolidae anatomy dorsally, especially *Offacolus kingi*, its ventral anatomy differs from this group in many aspects such us: a lower head tagmatization, reduced exopods, and absence of a division of the trunk into pre-abdomen and abdomen. However, the trunk exopods of *Sarotrocercus oblitus* may represent an early evolutionary step towards the opercula with the possible loss or strong reduction of the endopods. Confirmation of its chelicerates affinities is subject to further study.

Phylogenetic analyses

In this paper we decided to use two different phylogenetic analyses to assess the position of *Setapedites abundantis* based on two different matrices [27, 28]. However, each phylogenetic analysis may reflect some of the author's biases. To overcome as much as possible this problem, and trying to accommodate our findings into the broadest number of hypotheses about delicate topic regarding chelicerates evolution we provided different reiteration of each of our phylogenies. With the analyses shown in Supplementary Fig. 5, we tested the position of *Setapedites abundantis* in a matrix in which the chelicerates state of *Mollisonia plenovenatrix* is questioned and the same is true for Fig. 6 and Supplementary Figs. 6–8, reflecting the authors opinion on this topic. The tree in Supplementary Fig. 9 shows the results of the phylogenetic analyses coding for an Arachnopulmonata constraint into the phylogeny. Supplementary Fig. 10 shows the results of the phylogenetic analyses with *Mollisonia plenovenatrix* coded as in its original publication. Supplementary Figs. 11–15 instead, are reiterations of Supplementary Figs. 6–8, but excluding the artiopodans, since their relationship with chelicerates

are controversial. The results of all of these analyses, except for the one shown in Supplementary Figs. 14 and 15, support Offacolidae as a monophyletic group with a sister taxa position respectively to crown Euchelicerate, which is the main finding of this research. We have reported the different phylogenetic analyses, alongside the information with the different coding in Tab. 2

Definitions of tagmosis and related terms

Debates about the nature of the ancestral euchelicerate segmentation in the head and body hinge partly on the terminology used, and variable definitions for key terms such as tagmosis, tagma, pseudotagma, prosoma, opisthosoma, prosomal appendages and opisthosomal appendages. As stated in by Fusco and Minelli (2016, p. 212) in [29], "Although used to encapsulate the main features that characterize body architecture, the tagmata of a given animal often have boundaries whose positions are somehow disputable, depending on the definition of tagma adopted and on the interpretation of the body structures present in proximity to the putative boundaries themselves, in the context of the animal life history, morphology, development and evolution". Reviews of the definition of tagma in euchelicerates is available in Lamsdell (2013, p. 4) [28] which proposed to adopt the "term 'pseudotagmata' for units defined by differentiation of the tergites or sternites without an associated change in form or function of the appendages. By contrast true tagmata would be defined as regions of functional specialization, which in arthropods is predominantly mediated through modification or suppression of the appendages.". Conversely, pseudotagma (and so a cephalothorax) has been reported as "the units defined by differentiation of the tergites or sternites without an associated change in form or function of the appendages". This same definition has been adopted in a review of the tagmosis in chelicerates by Dunlop and Lamsdel (2017) [30]. The definition of tagma used in the phylogenies of Aria & Caron (2017, 2019) [5,27] is a mixture of the definition of tagma and pseudotagma, described as "somital head (as tagma I) defined by series of appendages and/or external segmentation" (Aria & Caron 2017 page 13 of supplemental information [5]; Aria & Caron 2019 Page 11 of supplemental information [27]). In this paper, we follow the definition found in Dunlop & Lansdell 2017 [30]. While this creates no controversies in the coding of characters in the matrix from Lamsdell 2013 [28], it may create some misunderstandings for our coding on the matrix from Aria & Caron (2019) [27]. For this last matrix we decided to do not update the coding for the character accounting for the tagmosis for two main reasons. Firstly, tagma and pseudotagma are just two different characters with no intrinsic value of being more or less informative, as far as is it clear what we are meaning by each of them. Secondly for the anatomy of *Habelia optata*, we do recognize that not only the pseudotagmatization of this species may incorporate seven tergites, but also that the seventh somite appendages may be, as described in Aria & Caron (2017) [5], functionally involved in the head tagma.

As such not having examinate the material ourselves we keep the exact coding of Aria & Caron (2019)[27] for this character in the matrix we used. Lastly, it should be noted that currently the anatomy of *Weinbergina*, used to sustain a seven-somite head tagma at the root of euchelicerates is considered by many as in need for a review [30,31].

SUPPLEMENTARY NOTES

Coding modified in Aria & Caron 2019's matrix

Offacolus

Character 68- Labrum in *Offacolus* from ? to 1

We assume the labrum of *Offacolus kingi* and *Setapedites abundantis* to be homologous with the one in euchelicerates.

Character 69- Position of labrum from ? to 0

Character 71- from ? to –

The above changes have been made on all the iterations of analyses on this matrix (Supplementary data 1–3).

Mollisonia

Character 123- Stenopodous exopod type coded as both ? and 1 (respectively Supplementary data 1 and 2)

We ran two Bayesian analyses, one with this character state coded as ?, resulting in the trees of Supplementary Figs. 5–8 and another where this state is kept as 1 as originally coded in Aria & Caron (2019) and resulting in Supplementary Figs. 10 and 11. This is because, in our opinion, this structure (described from a single exopod in one specimen in Aria & Caron 2019) is difficult to interpret in detail but seems to have a different structure to that seen in *Setapedites abundatis*.

Coding modified in Lamsdell 2013's matrix

Weinbergina

Character 69, 70, 71, 72, 73, 100- modified according to Selden et al 2015

Mollisonia

Character 69- Exopod on second post-antennular limb coded as both 3 and 2 (respectively Supplementary data 4 and 5).

We ran two Bayesian analyses, one with character state 3 (reduced state of prosomal exopod), resulting in the trees of Fig. 6 and Supplementary Figs. 6–8, and another where this state is changed

to 2 (pediform exopod), resulting in the trees of Supplementary Fig. 11. These two different codings reflect different interpretations of *Mollisonia* cephalic exopods in comparison with offacolids. The two different codings however do not change the result of the analyses for the target group.

Character 110 modified

We added to character 110 (originally five states) a sixth state for the bifurcate tip of telson and coded it present in *Dibasterium durgae*, *Setapedites abundantis* and *Offacolus kingi*

Fuxianhuia protensa and *Willwerathia laticeps* have been removed, *Habelia optata* and *Mollisonia plenovenatrix* added.

Additional considerations

The above changes have been made on all the iterations of analyses on this matrix (Supplementary data 4,5,7 and 9).

The matrix used for the parsimony analysis, Supplementary data 6, has the same coding as Supplementary data 4.

We also tested the effects of removing the artiopodans (*Olenoides serratus*, *Emeraldella brocki*, and *Sidneyia inexpectans*) from the matrix resulting in Supplementary Figs. 12–15, Supplementary Data 7–9.

See also table 2 for a list of all the different phylogeny reiterations.

SUPPLEMENTARY FIGURES

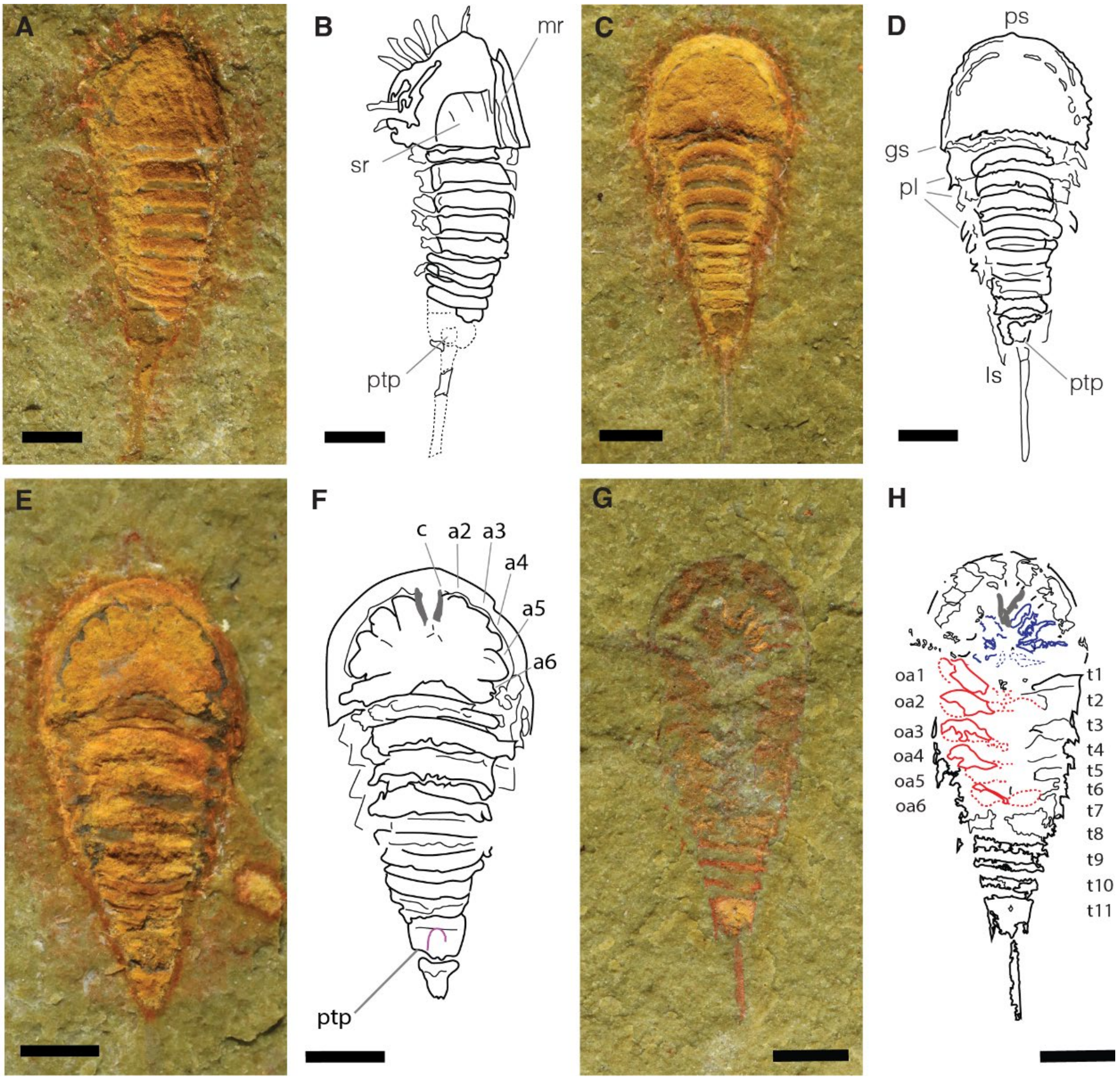

Supplementary Figure 1. Additional photographs of specimens of *Setapedites abundantis gen. et sp. nov.*
(A and B) MGL.102952 and interpretative drawing, articulated specimen in dorso-lateral view (horizontally mirrored).
(C and D) MGL. 102800b and interpretative drawing, articulated specimen in dorsal view.
(E and F) MLG.102902 and interpretative drawing, articulated specimen in dorsal view with prosoma open, showing appendages.
(G and H) MGL. 102469 and interpretative drawing, articulated specimen in ventral view showing the opisthosomal appendages.
a2–6, prosomal appendages 2–6; gs, genal spine; ls, spine lateral to the telson; mr, median ridge; oa1–6, opisthosomal appendages 1–6; pl, pleura; ps, prosomal spine; ptp, pretelsonic process; sr, sunken region; t1–11, tergites 1–11. Chelicerae are highlighted in grey, endopods in blue and opisthosomal exopods in red.
Scale bars 1 mm.

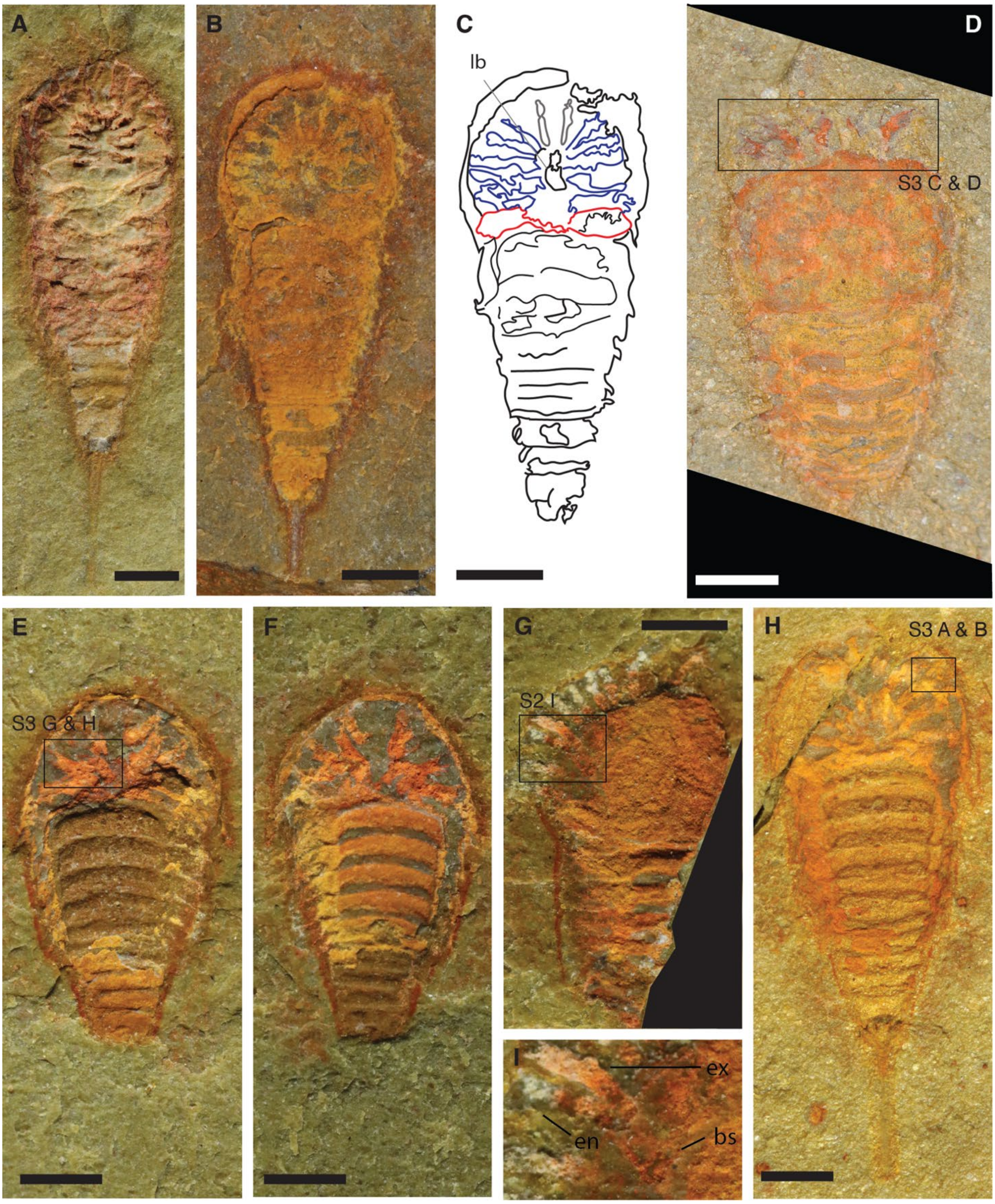

Supplementary Figure 2. Additional photographs of figured specimens of *Setapedites abundantis gen. et sp. nov.*
(A) MGL.107741, holotype, photographed under normal lightning, Fig. 3.
(B and C) YPM IP 517932 and interpretative drawing, part, Fig. 2A and 2B.
(D) MGL. 102247a, counterpart.
(E) MGL.102634, counterpart, Fig. 2G and 2H, full specimen.
(F) MGL.102634, part.
(G) MGL.102934, Fig. 2E and 2F, full specimen.
(H) MGL.102800a, Fig. 2J and 2K, full specimen.
(I) MGL.102934, Fig. 2E and 2F, detail of appendage (exopod, endopod and common basipodite).
en, endopod; ex, exopod; bs, basipodite; lb, labrum.
Chelicerae are highlighted in grey, endopods in blue and opisthosomal exopods in red. Scale bars 1 mm.

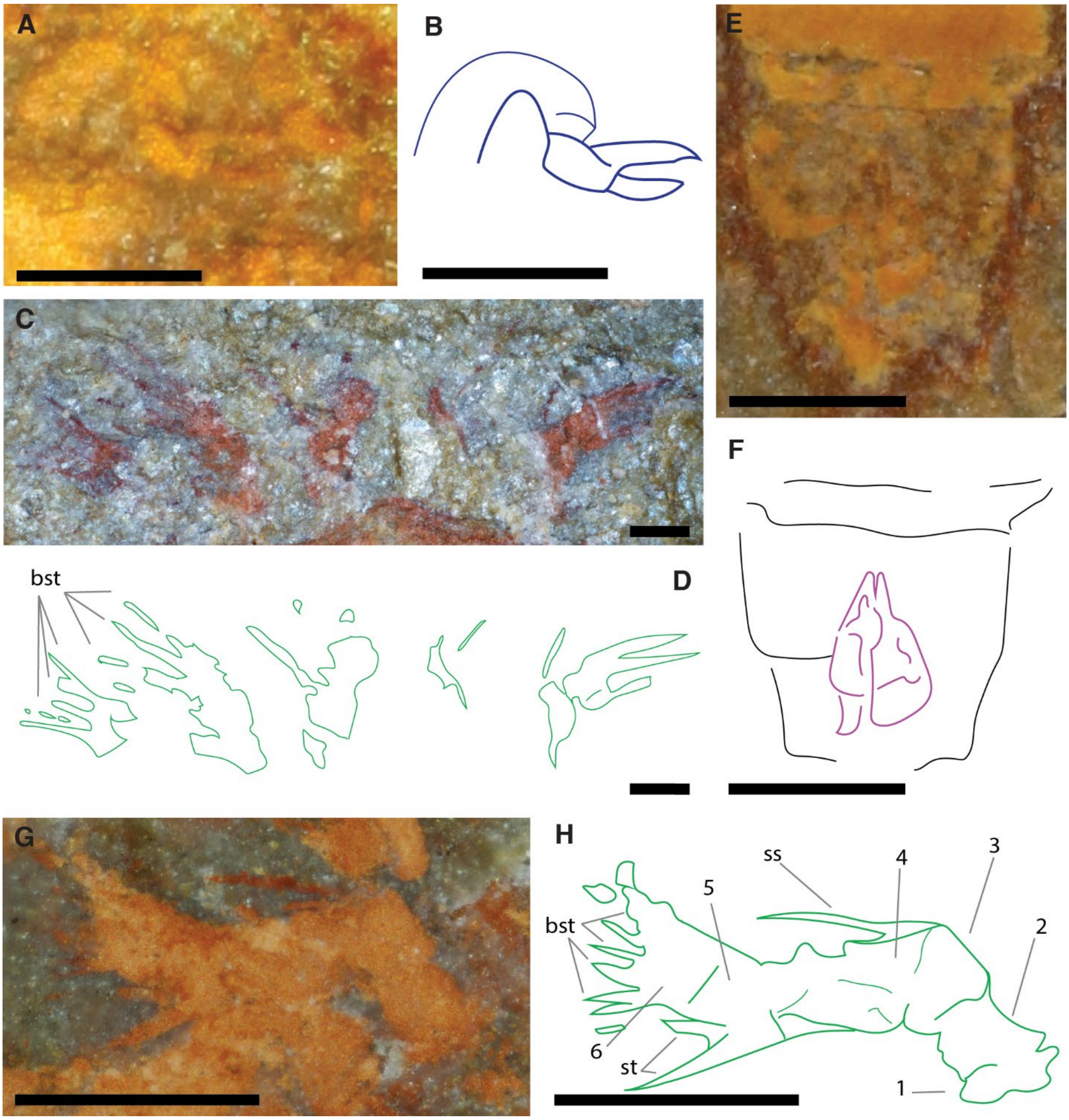

Supplementary Figure 3. Additional photographs of figured specimens of *Setapedites abundantis gen. et sp. nov.*
(A and B) MGL.102800a and interpretative drawing, Fig. 2J and 2K and Supplementary Fig. 2H, chelate endopod detail
(C and D) MGL. 1022 47a and interpretative drawing, Supplementary Fig. 1D, prosomal exopod anatomy detail.
(E and F) YPM IP 517932c (counterpart) and interpretative drawing, Fig. 2A and 2B, pretelsonic process detail
(G and H) MGL.102634 and interpretative drawing, Fig. 2G and 2H and Supplementary Fig. 2E, prosomal exopod anatomy detail
bst, brush-like setae; ss, single setae; st, pair of setae; 1–6, podomeres. Endopods are highlighted in in blue, exopods in green and the pretelsonic process in purple.
Scale bars 0.2 mm A–D, 0.5 mm E–H.

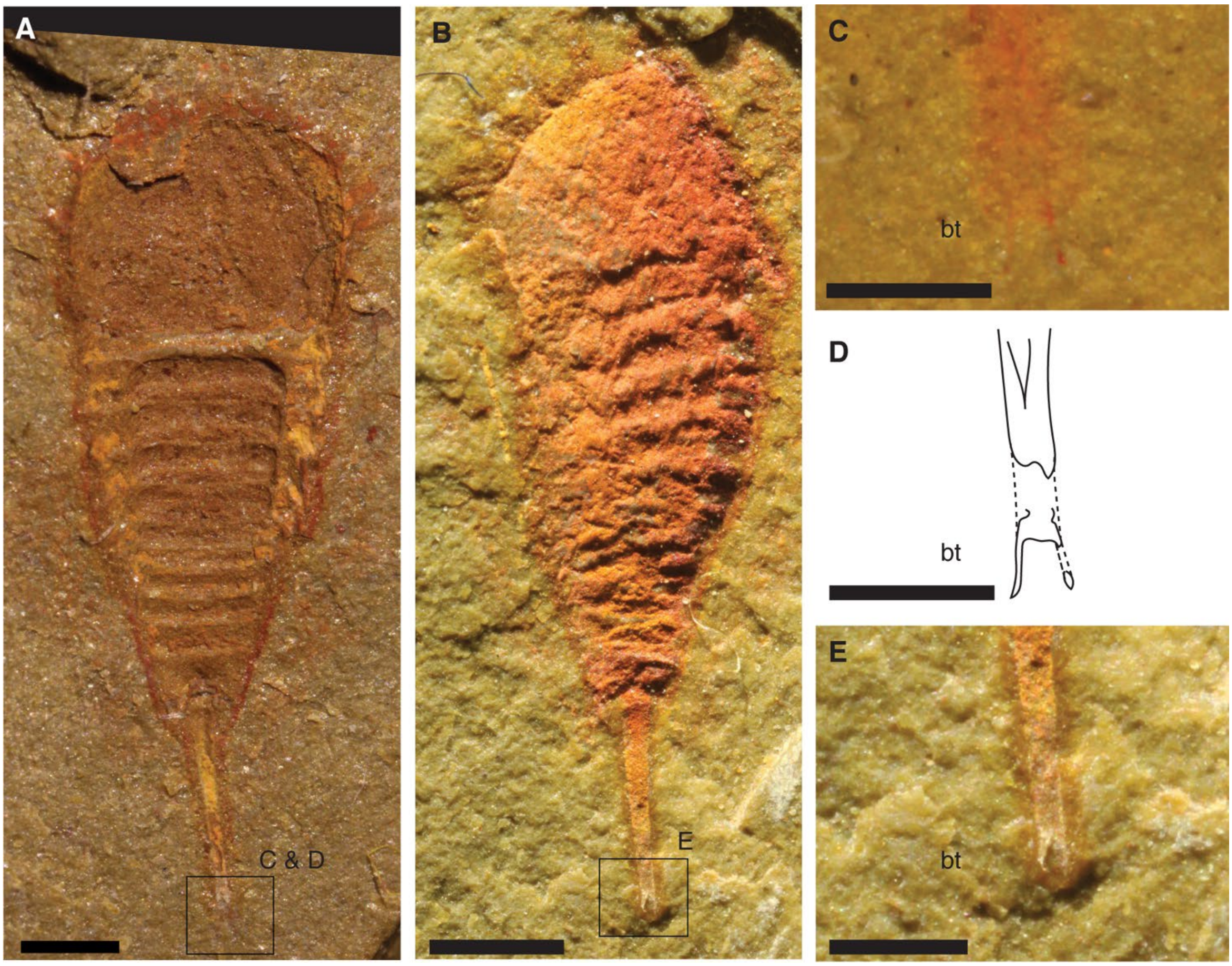

Supplementary Figure 4. Telson morphology of *Setapedites abundantis gen. et sp. nov.*
(A) MGL. 102899, counterpart, Fig. 1A and B, a specimen with tip of the telson preserved.
(B) MGL.102690, articulated specimen in dorsal view.
(C and D) MGL.102899, close-up and drawing of the posterior region of the abdomen and telson.
(D) MGL.102690, close-up of the posterior region of the abdomen and telson.
bt, bifurcate telson.
Scale bars 1 mm A and B, 500 µm C–E.

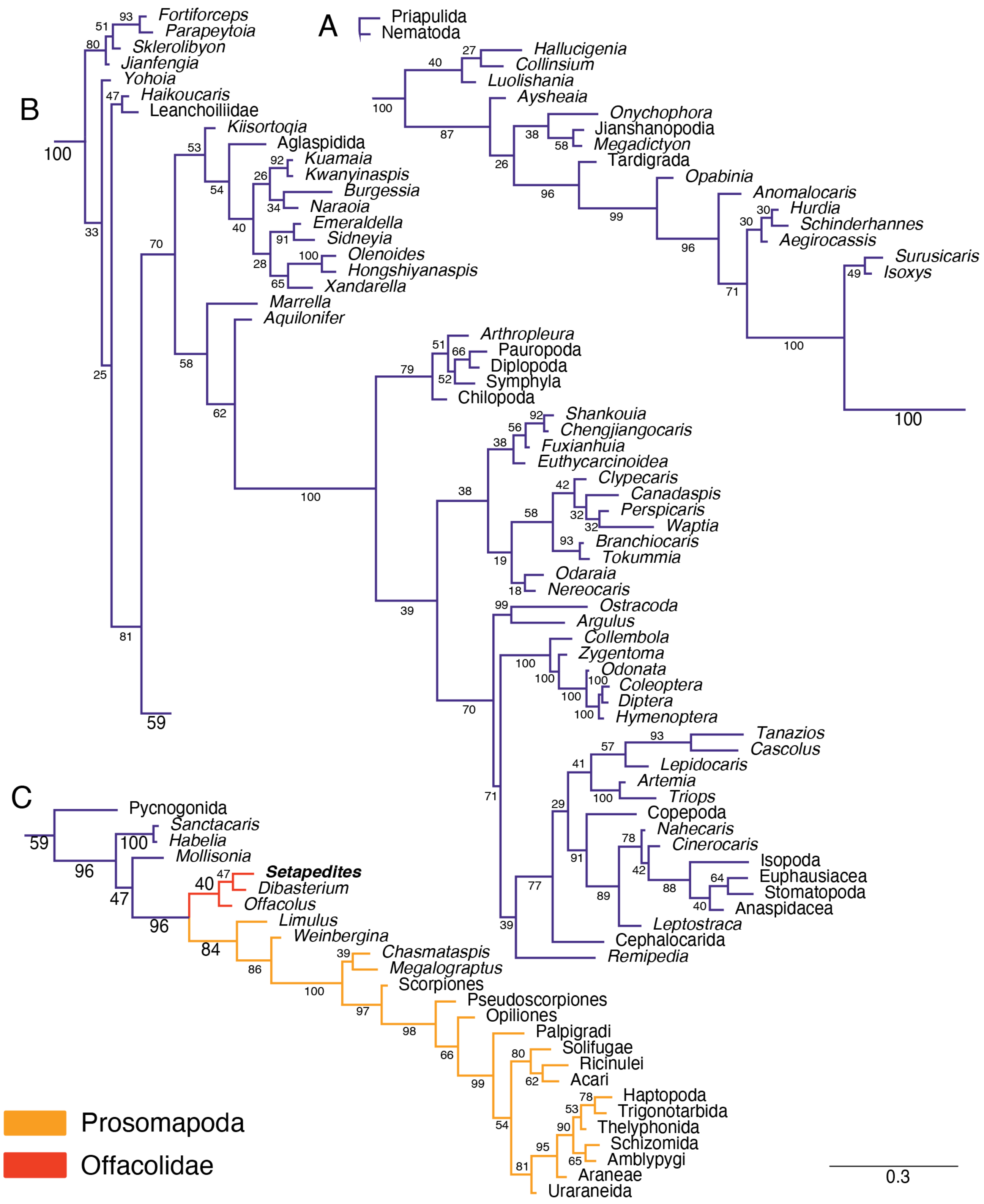

Supplementary Figure 5. Phylogenetic position of *Setapedites abundantis gen. et sp. nov.* among arthropods. Extended majority rule tree of a Bayesian analysis. Numbers next to nodes are posterior probabilities when <100. Matrix modified from Aria and Caron (2019) as reported in the supplementary information. A, root section of the tree; B, middle section of the tree, C euchelicerates section of the tree.
Branches of the Prosomapoda and Offacolidae are coloured in orange and red, respectively, and the rest of the tree is coloured in purple.

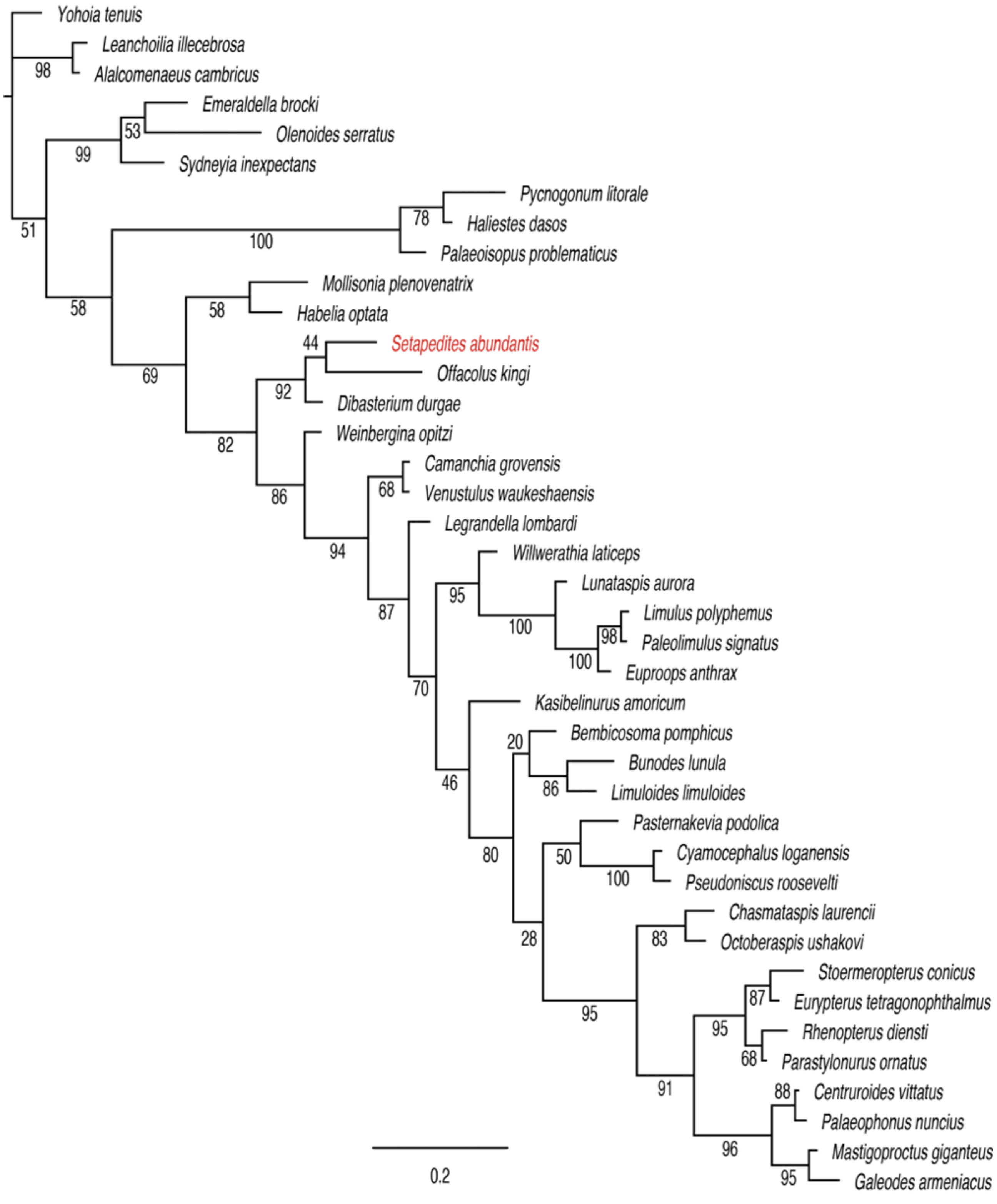

Supplementary Figure 6. Phylogenetic position of *Setapedites abundantis gen. et sp. nov.* among euchelicerates. Extended majority rule tree of a Bayesian analysis used for Fig. 6. Numbers next to nodes are posterior probabilities when <100. Matrix modified from Lamsdell (2013). Coding for *Mollisonia* and *Habelia* based on Aria & Caron 2019 and modified as reported in the supplementary information.

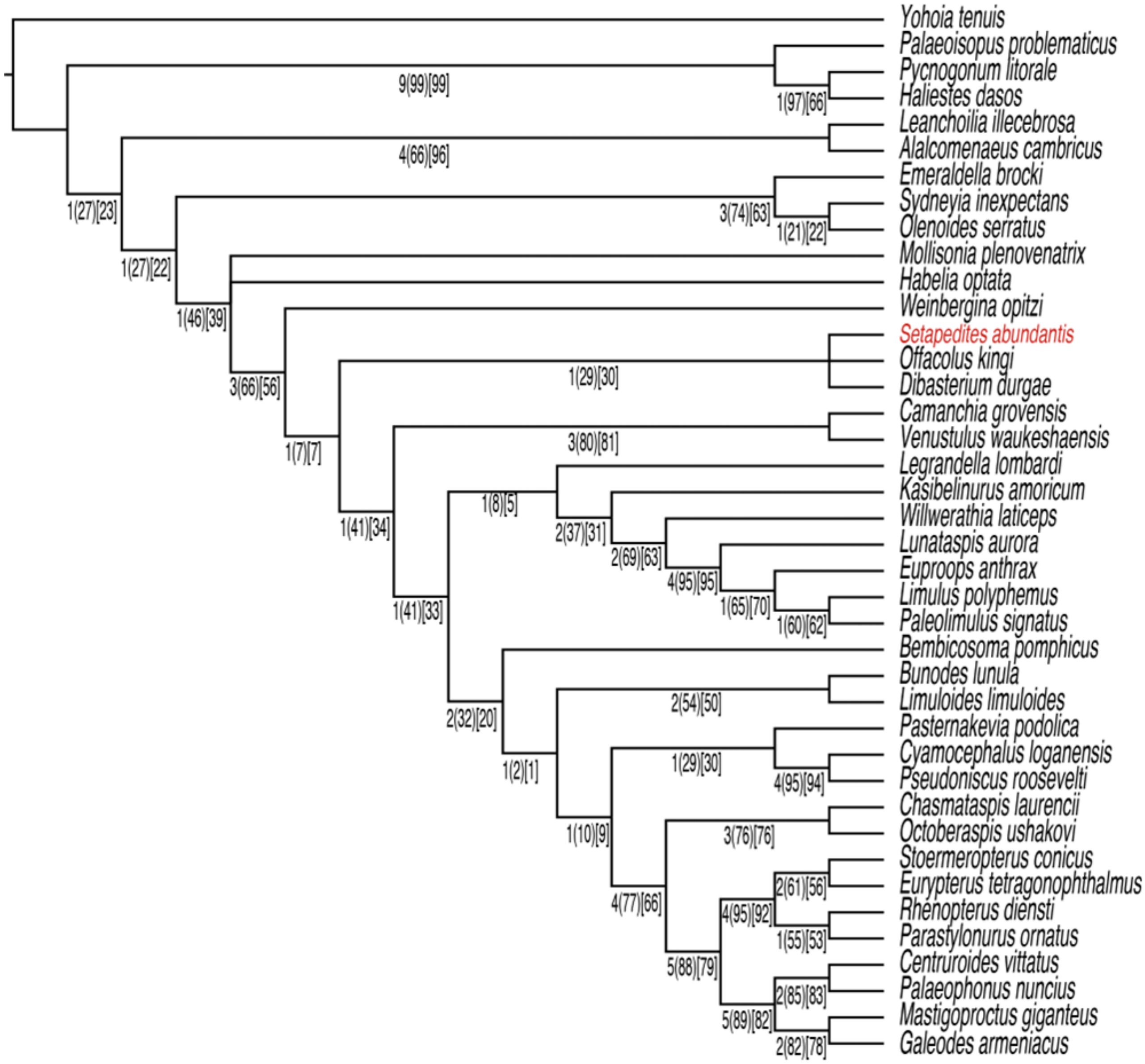

Supplementary Figure 7. Phylogenetic position of *Setapedites abundantis gen. et sp. nov*. among euchelicerates. Parsimony analyses result of the strict consensus of 4 most parsimonious trees. Phylogenetic analysis performed using random addition sequences followed by branch swapping with 100 000 repetitions, all characters unordered and of equal weight followed by Jacknife (33% deletion, 1000 repetitions) and Bootstrap (50% deletion, 1000 repetitions). Bremer support is shown with no brackets, Jacknife support is shown between round brackets and Bootstrap support is shown between square brackets. Matrix modified from Lamsdell (2013).

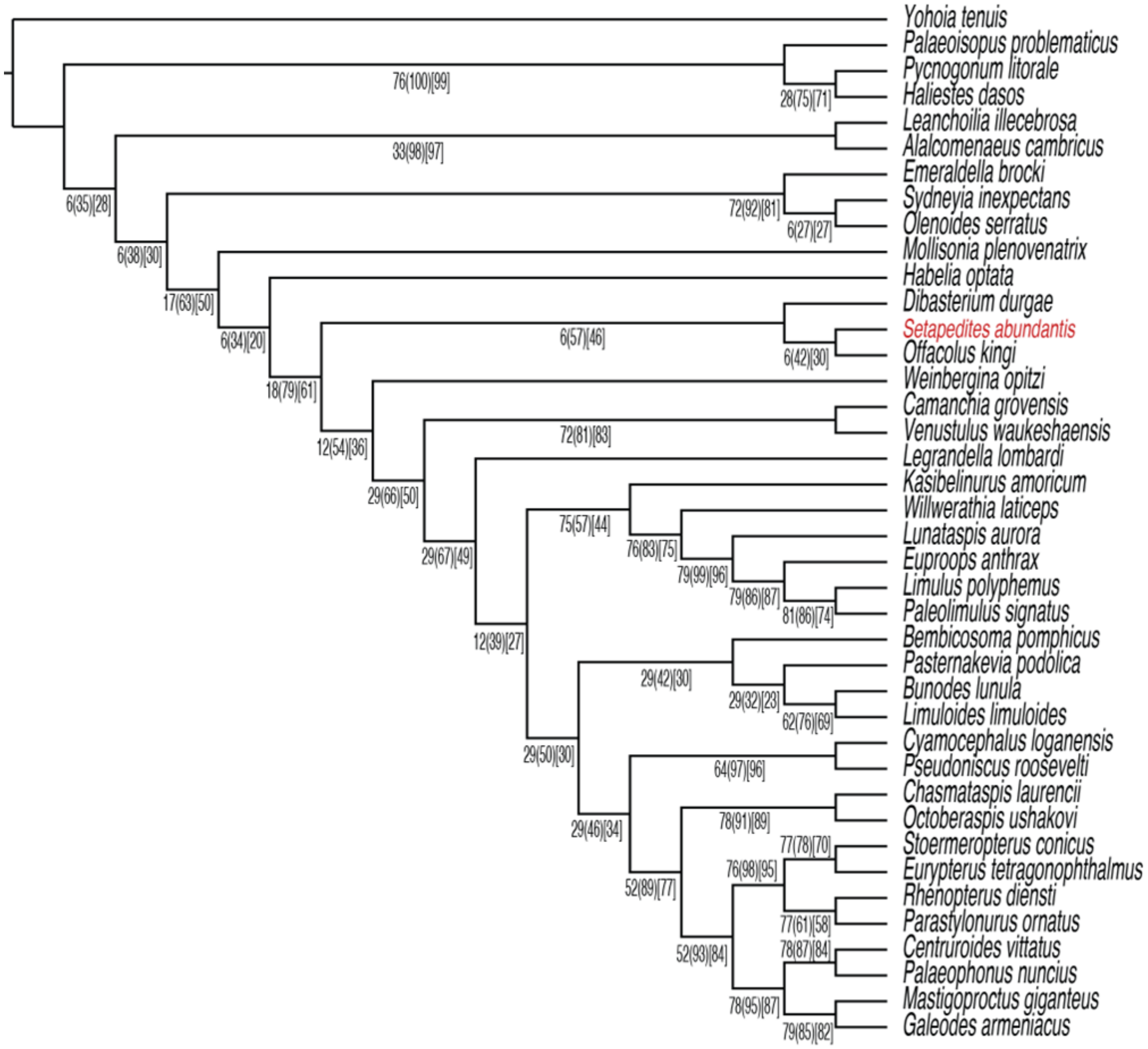

Supplementary Figure 8. Phylogenetic position of *Setapedites abundantis gen. et sp. nov.* among euchelicerates. Parsimony analyses single most parsimonious trees. Phylogenetic analysis performed using random addition sequences followed by branch swapping with 100 000 repetitions, implied weight 12K, followed by Jacknife (33% deletion, 1000 repetitions) and Bootstrap (50% deletion, 1000 repetitions). Bremer relative support is shown with no brackets, Jacknife support is shown between round brackets and Bootstrap support is shown between square brackets. Matrix modified from Lamsdell (2013).

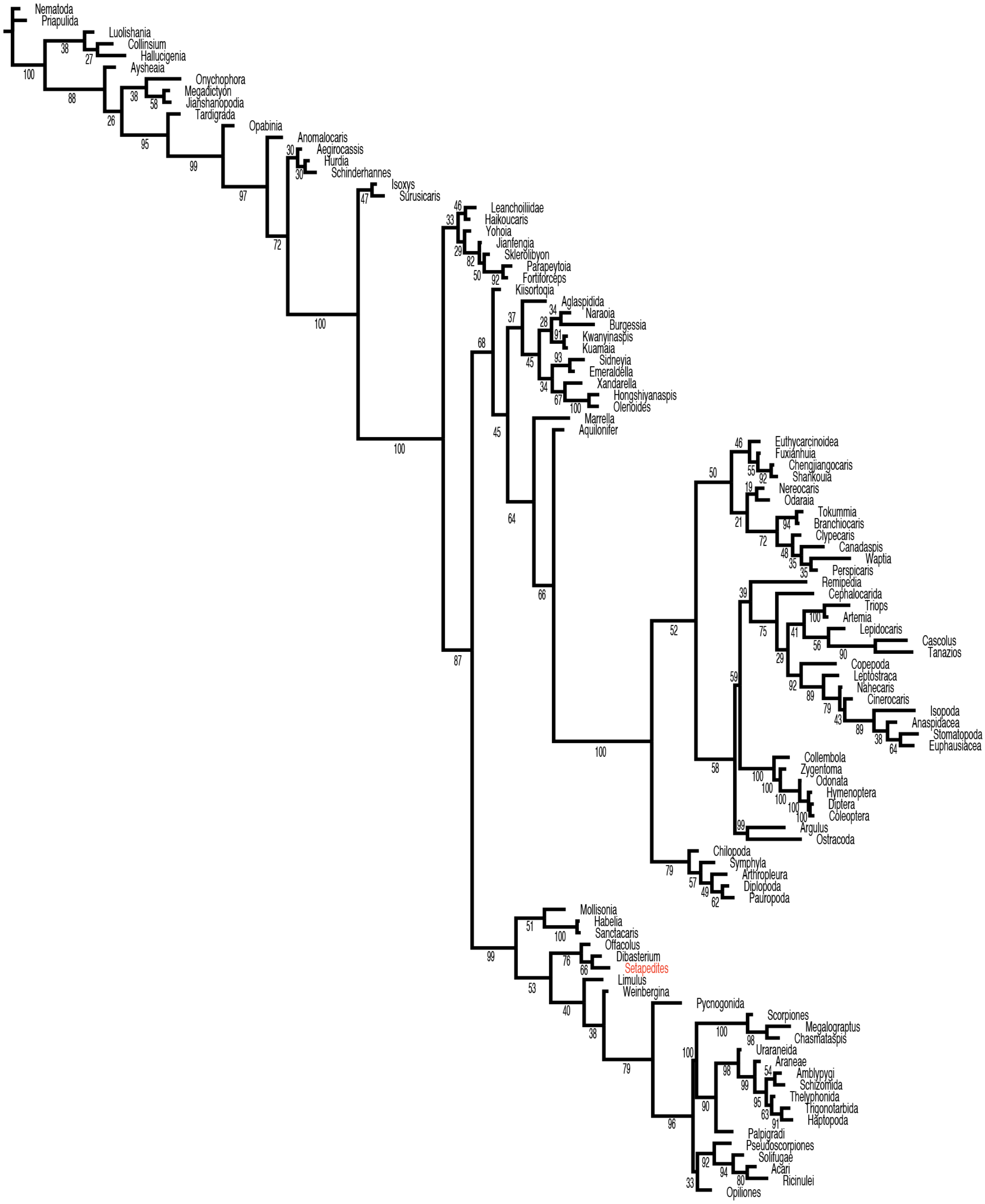

Supplementary Figure 9. Phylogenetic position of *Setapedites abundantis gen. et sp. nov.* among arthropods. Extended majority rule tree of a Bayesian analysis using the Arachnopulmonata constraint. Numbers next to nodes are posterior probabilities when <100. Matrix modified from Aria and Caron (2019).

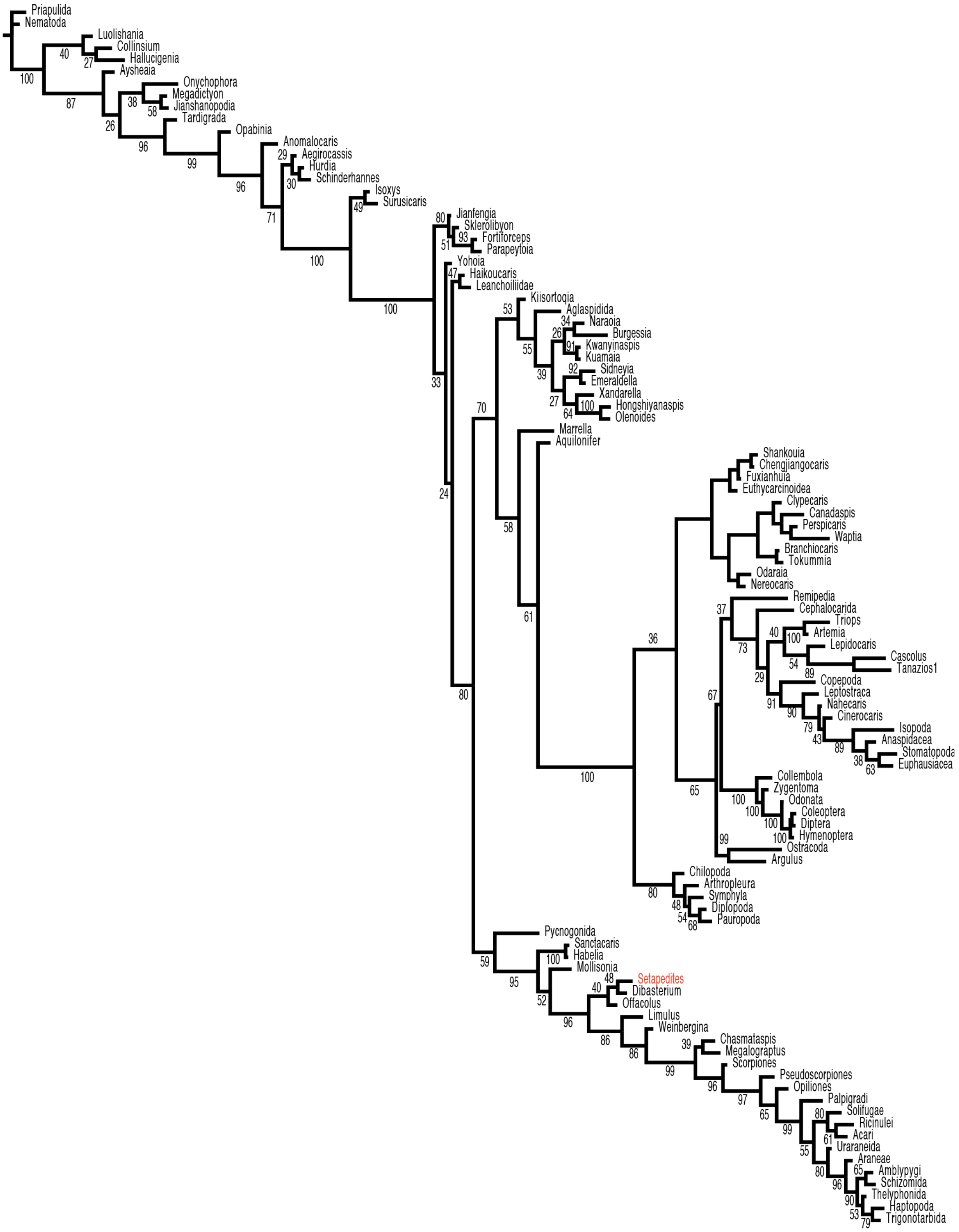

Supplementary Figure 10. Phylogenetic position of *Setapedites abundantis gen. et sp. nov.* among arthropods. Extended majority rule tree of a Bayesian analysis. Numbers next to nodes are posterior probabilities when <100. Matrix modified from Aria and Caron (2019) with the addition of *Setapedites* but preserving the coding for *Mollisonia*.

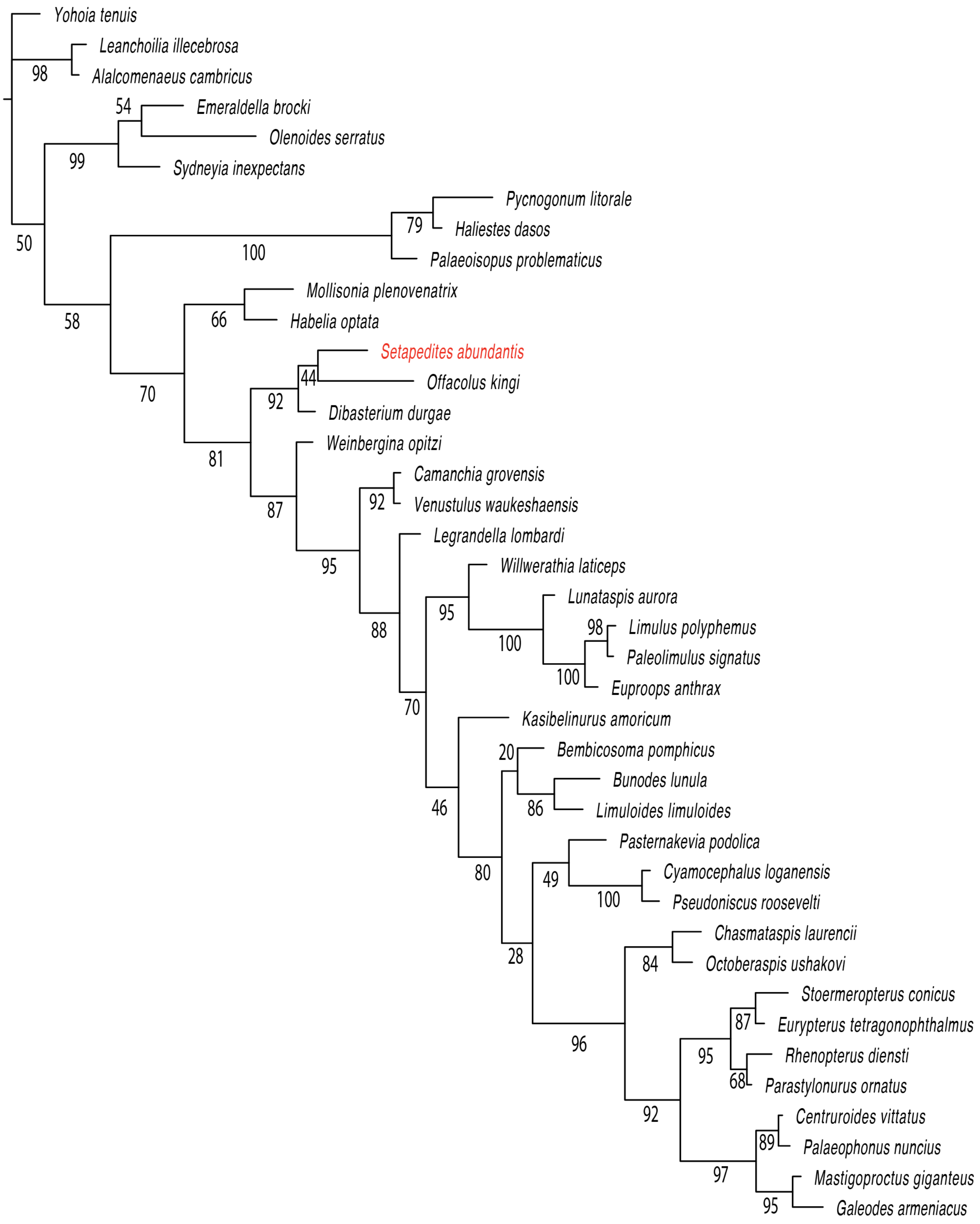

Supplementary Figure 11. Phylogenetic position of *Setapedites abundantis gen. et sp. nov.* among euchelicerates. Extended majority rule tree of a Bayesian analysis used for Fig. 6 but preserving the coding for *Mollisonia* based on Aria & Caron 2019. Numbers next to nodes are posterior probabilities when <100. Matrix modified from Lamsdell (2013).

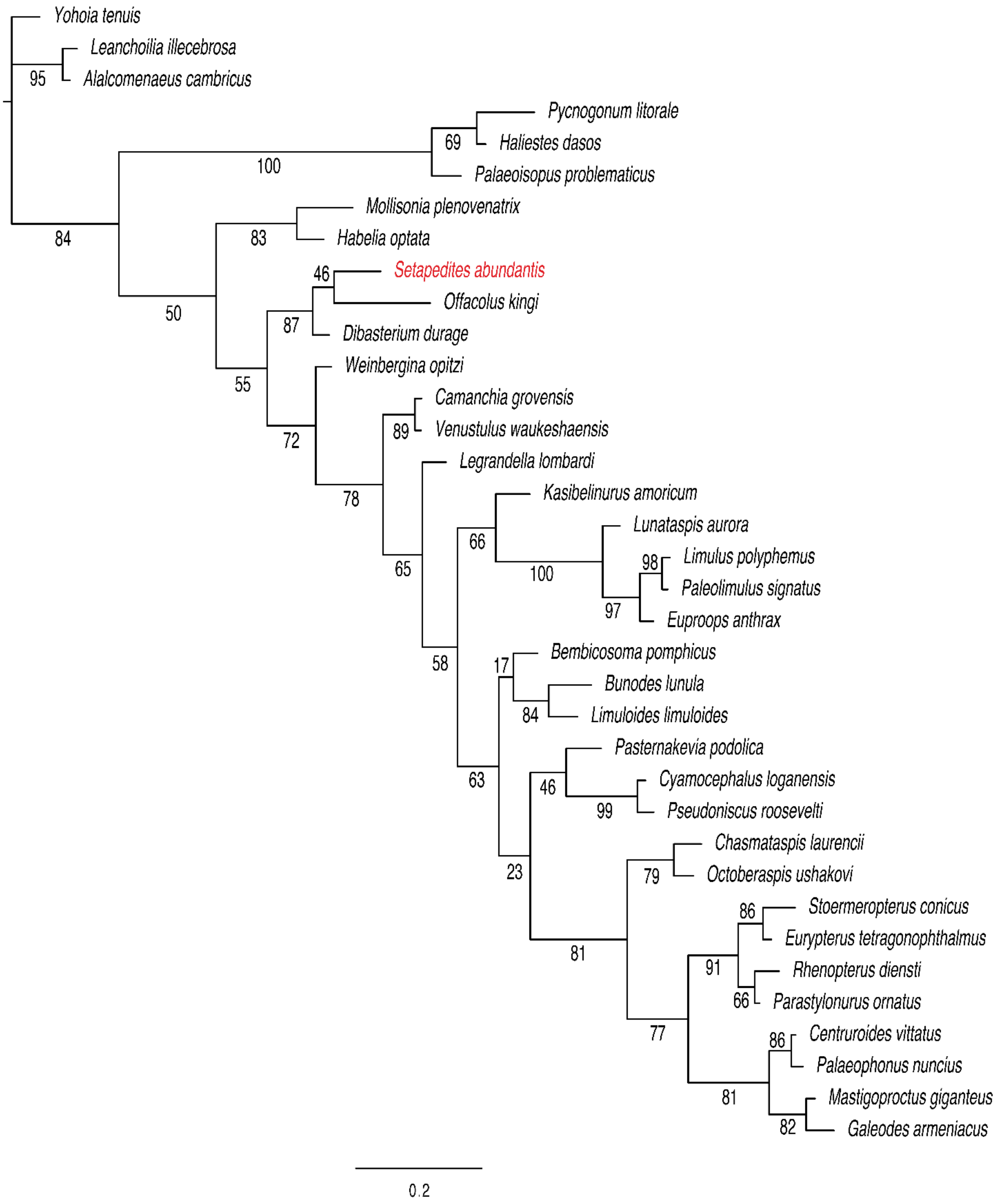

Supplementary Figure 12. Phylogenetic position of *Setapedites abundantis gen. et sp. nov.* among euchelicerate s excluding the artiopodans from the outgroups. Extended majority rule tree of a Bayesian analysis using the same matrix and coding as for Fig. 6 but also excluding the artiopodans. Numbers next to nodes are posterior probabilities when <100. Matrix modified from Lamsdell (2013). Coding for *Mollisonia* and *Habelia* based on Aria & Caron 2019 and modified as reported in the supplementary information.

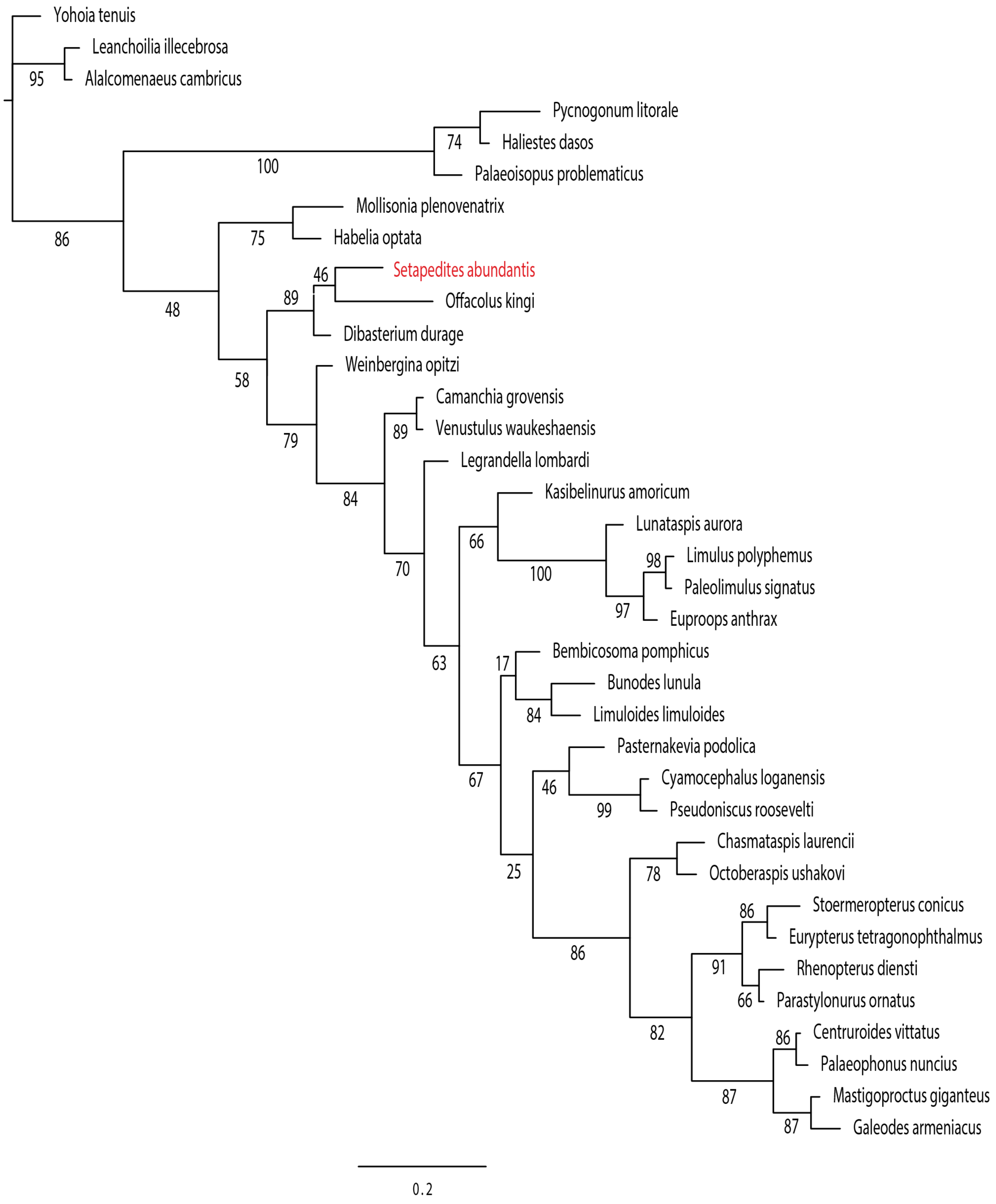

Supplementary Figure 13. Phylogenetic position of *Setapedites abundantis gen. et sp. nov.* among euchelicerate s excluding the artiopodans from the outgroups.
Extended majority rule tree of a Bayesian analysis using the same matrix and coding as for Supplementary Fig. 11 but also excluding the artiopodans. Numbers next to nodes are posterior probabilities when <100. Matrix modified from Lamsdell (2013).

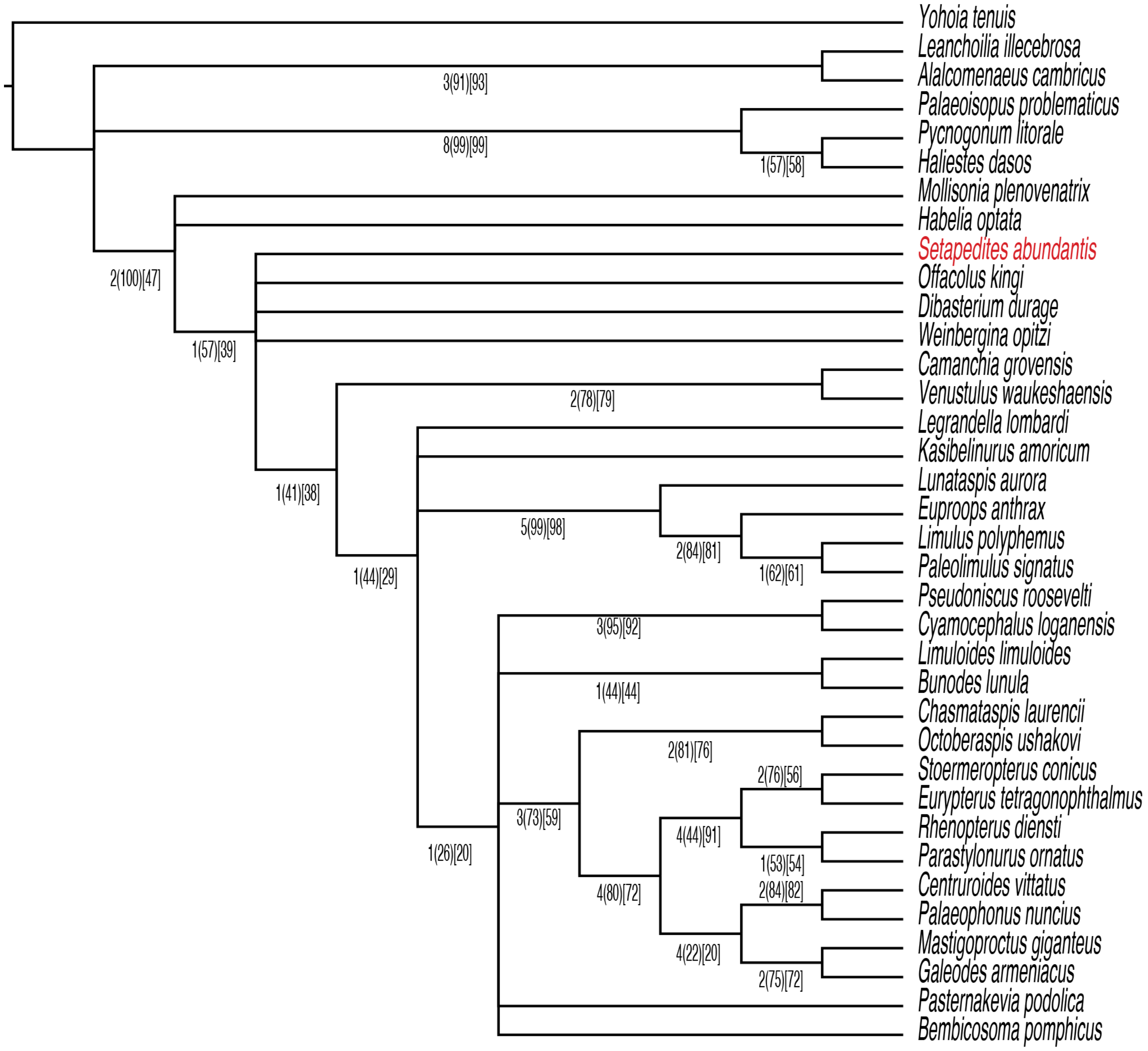

Supplementary Figure 14. Phylogenetic position of *Setapedites abundantis gen. et sp. nov*. among euchelicerates excluding the artiopodans from the outgroups.

Parsimony analyses result of the strict consensus of 30 most parsimonious trees. Phylogenetic analysis performed using random addition sequences followed by branch swapping with 100 000 repetitions, all characters unordered and of equal weight followed by Jacknife (33% deletion, 1000 repetitions) and Bootstrap (50% deletion, 1000 repetitions). Bremer support is shown with no brackets, Jacknife support is shown between round brackets and Bootstrap support is shown between square brackets. Matrix modified from Lamsdell (2013).

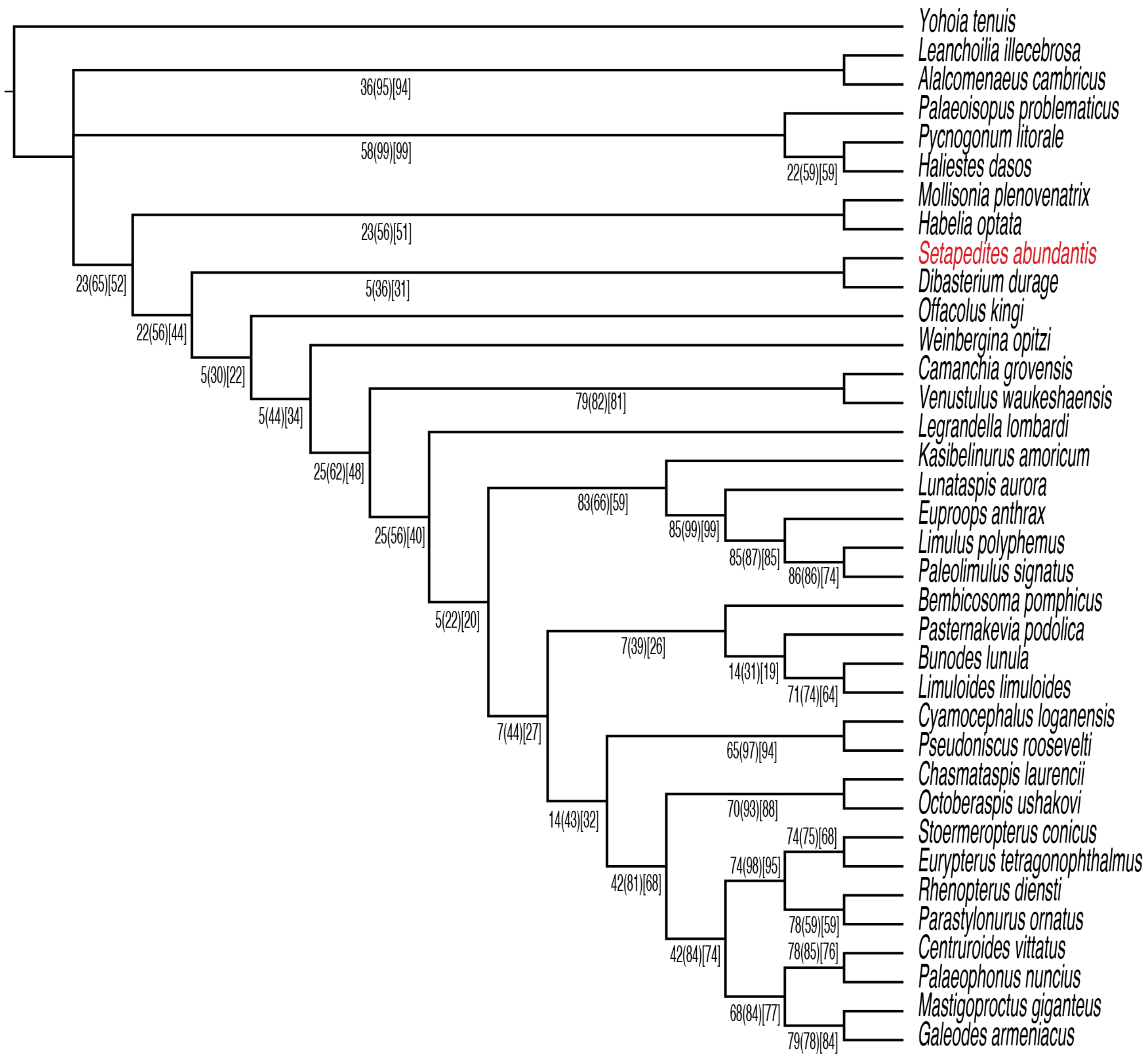

Supplementary Figure 15. Phylogenetic position of *Setapedites abundantis gen. et sp. nov.* among euchelicerates excluding the artiopodans from the outgroups.

Parsimony analyses single most parsimonious trees. Phylogenetic analysis performed using random addition sequences followed by branch swapping with 100 000 repetitions, implied weight 12K, followed by Jacknife (33% deletion, 1000 repetitions) and Bootstrap (50% deletion, 1000 repetitions). Bremer relative support is shown with no brackets, Jacknife support is shown between round brackets and Bootstrap support is shown between square brackets. Matrix modified from Lamsdell (2013).

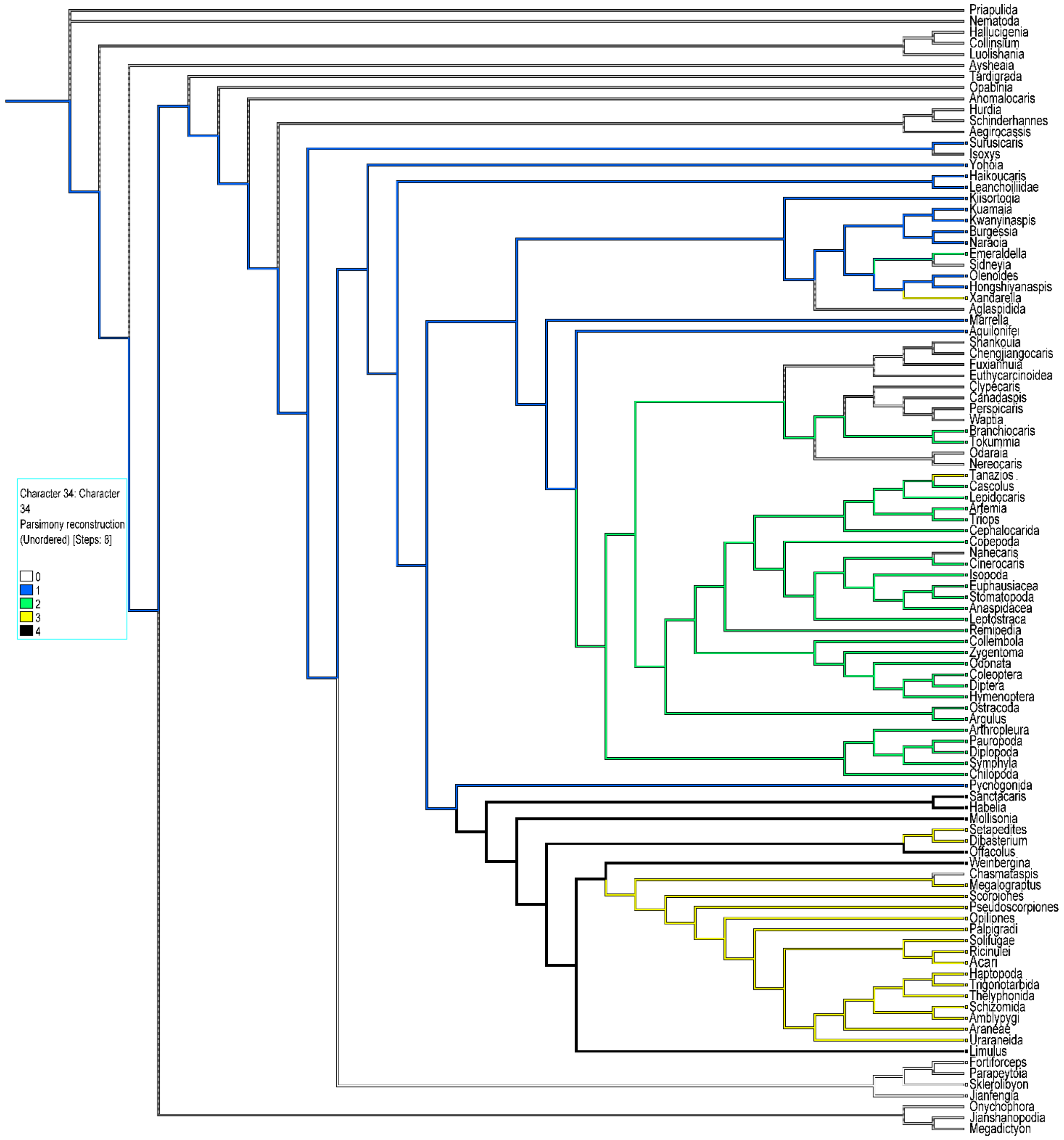

Supplementary Figure 16. Reconstructed ancestral state for the cephalothorax pseudotagmosis (referred to as prosoma in Aria & Caron 2017, 2019) based on the extended majority rule tree of a Bayesian analysis shown in Supplementary Fig. 5.

N.B. In Aria & Caron 2017, 2019 the ocular somite is counted as somite 1 while in this work, according with Dunlop & Lamsdell 2017 is counted as somite 0. In the original matrix the character is coded as “Somites defining anteriormost tagma and is coded as 0= four somite; 1. = five somite; 2. = six somite; 3= seven somite; 4= eight somite. In the present work a tagma is not defined by the dorsal anatomy. According with this reconstruction, a seven-somite cephalothorax is plesiomorphic to Euchelicerate with two reversals, at the internal node of Offacolidae and at the root of Dekatriata.

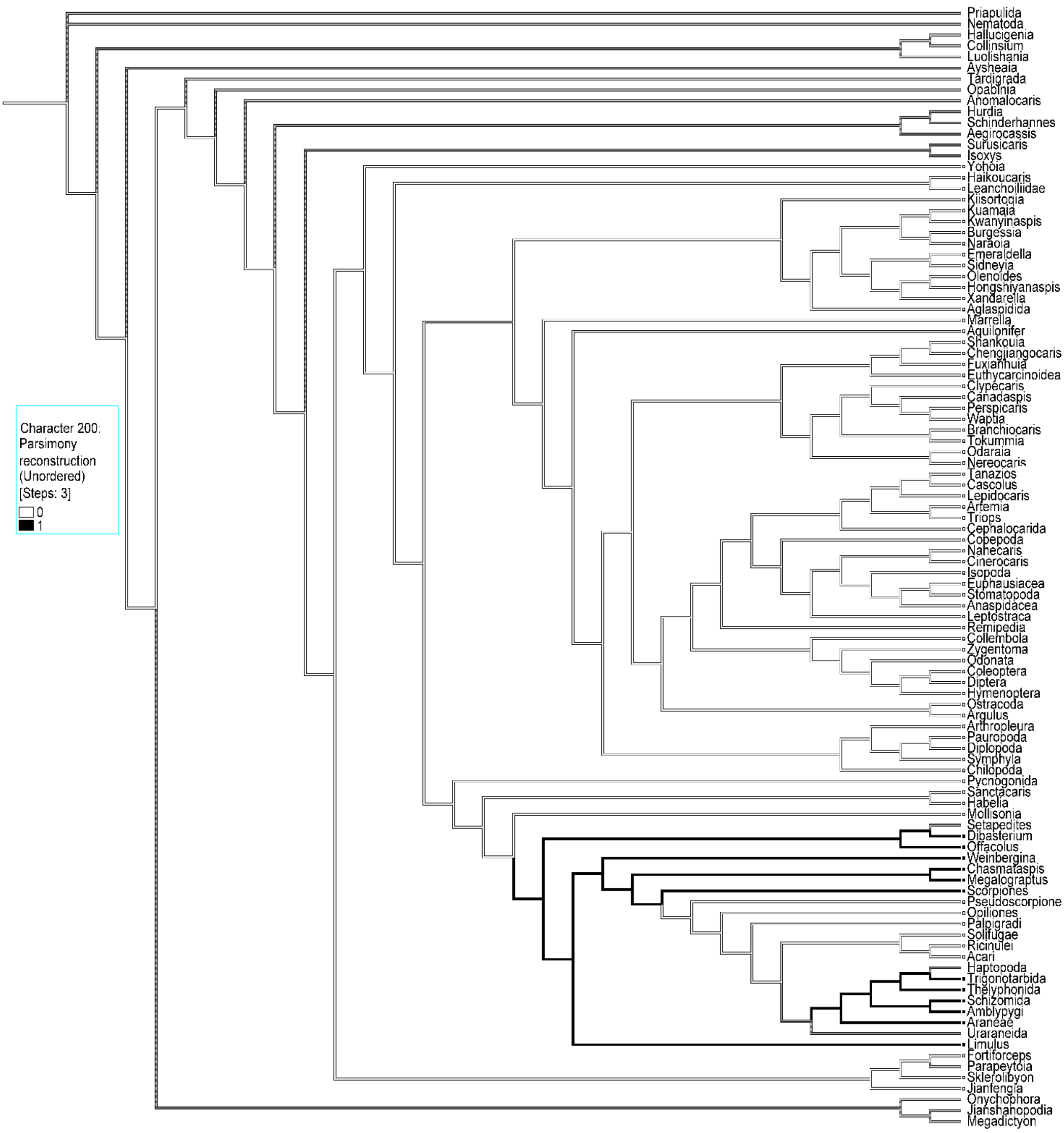

Supplementary Figure 17. Reconstructed ancestral state for the opercula based on the extended majority rule tree of a Bayesian analysis shown in Supplementary Fig. 5.
In the original matrix the character is coded as “Post-cephalic appendages covered by sclerotic plates (opercula) 0= Absent; 1= Present. Note that in the present work a tagma is not defined by the dorsal anatomy. According with this reconstruction a seven-somite prosoma is found only in Lower stem euchelicerates. According with this reconstruction the presence of opercula is expected in *Setapedites*.

SUPPLEMENTARY TABLES

Supplementary Table 1. Measurements of *Setapedites abundantis gen. et sp. nov.* material.
Total length and maximum width of the specimens figured in this study. Measurements not available for variables not fully preserved are marked as "/".

Specimen	Museum	Figure(s)	Total length	Maximum width
MGL.107741 (holotype)	MGL	Figure 3A–D and S2a	6.5 mm	2.9 mm
YPM IP 517932	YPM	Figure S2b and 2c	5.81 mm	2.23 mm
YPM IP 517932c	YPM	Figure 2A–D and S3e and 3f	5.99 mm	2.39 mm
MGL.102902	MGL	Figure S2e and 2f	5.84 mm	2.68 mm
MGL.102934	MGL	Figures 2E and 2F and S2g	/	/
MGL.102634	MGL	Figures 2G and 2H and S1e and 2f and S3g and 3h	/	/
MGL.102899	MGL	Figure 1A and 1B and S4a, c and d	5.73 mm	2.61 mm
MGL.102828	MGL	Figure 1C and 1D	4.33 mm	2.39 mm
MGL.102800a	MGL	Figures 2I and 2J S2h and S3a and 3b	5.50 mm	/
MGL.102800b	MGL	Figure S1c and 1d	4.85 mm	2.29 mm
MGL.102952	MGL	Figure S1a and 1b	5.07 mm	/
MGL.102872	MGL	Figure 1I and 1J	5.29 mm	/
MGL.102690	MGL	Figure S4B and 1E	5.30 mm	/
MGL.102637	MGL	Figure 4	/	/
MGL.102469	MGL	Figure S2g and 2h	/	/
MGL.102247a	MGL	Figure S2d and S3c and 3d	5.45 mm	2.51 mm

Supplementary Table 2. Edits performed on all different iterations of the phylogenetic analyses resulting in the trees shown in Supplementary Figs. 5–15.
Figures annotated as SX in column 1 refer to the respective Supplementary Fig. X.

Figures	Methods	Supplementary Data	Matrix	Edits
S5	Bayesian	1	Aria & Caron (2019)	*Mollisonia plenovenatrix* Character 123 coded as ? (chelicera uknown)
S6, 6	Bayesian	4	Lamsdell (2013)	Addition of *Mollisonia plenovenatrix* (Character 69 coded as 3) and *Habelia optata*, removed *Fuxianhuia protensa* and *Willwerathia laticeps*
S7	Parsimony E.W.	6	Lamsdell (2013)	Addition of *Mollisonia plenovenatrix* (Character 69 coded as 3) and *Habelia optata*, removed *Fuxianhuia protensa* and *Willwerathia laticeps*
S8	Parsimony 12K	6	Lamsdell (2013)	Addition of *Mollisonia plenovenatrix* (Character 69 coded as 3) and *Habelia optata*, removed *Fuxianhuia protensa* and *Willwerathia laticeps*
S9	Bayesian	3	Aria & Caron (2019)	Arachnopulmonata constraint
S10	Bayesian	2	Aria & Caron (2019)	*Mollisonia plenovenatrix* Character 123 coded as 1 (chelicera present)
S11	Bayesian	5	Lamsdell (2013)	Addition of *Mollisonia plenovenatrix* (Character 69 coded as 2) and *Habelia optata*, Remove *Fuxianhuia protensa* and *Willwerathia laticeps*
S12	Bayesian	7	Lamsdell (2013)	Addition of Mollisonia plenovenatrix (Character 69 coded as 3) and *Habelia optata*, removed *Fuxianhuia protensa* and *Willwerathia laticeps and artiopodans.*
S13	Bayesian	8	Lamsdell (2013)	Addition of *Mollisonia plenovenatrix* (Character 69 coded as 2) and *Habelia optata*, removed *Fuxianhuia protensa*, *Willwerathia laticeps* and artiopodans
S14	Parsimony E.W.	9	Lamsdell (2013)	Addition of *Mollisonia plenovenatrix* (Character 69 coded as 3) and *Habelia optata*, removed *Fuxianhuia protensa*, *Willwerathia laticeps* and artiopodans
S15	Parsimony 12K	9	Lamsdell (2013)	Addition of *Mollisonia plenovenatrix* (Character 69 coded as 3) and *Habelia optata*, removed *Fuxianhuia protensa*, *Willwerathia laticeps* and artiopodans

Supplementary Table 3. Chronological data for the 39 taxa of the phylogenetic tree used to create the chronogram of Figure 6.

	FAD	LAD
Fuxianhuia protensa	520	513
Yohoia tenuis	513	505
Leanchoilia illecebrosa	520	513
Alalcomenaeus cambricus	513	505
Sydneyia inexpectans	513	505
Emeraldella brocki	513	505
Olenoides serratus	513	505
Palaeoisopus problematicus	409.1	402.5
Pycnogonum litorale	0	0
Haliestes dasos	433.4	427.4
Habelia optata	513	501
Mollisonia plenovenatrix	513	501
Setapedites abundantis	485.4	477.7
Offacolus kingi	433.4	427.4
Dibasterium durgae	433.4	427.4
Weinbergina opitzi	409.1	402.5
Camanchia grovensis	433.4	427.4
Venustulus waukeshaensis	443.4	428
Legrandella lombardi	412.3	409.1
Kasibelinurus amoricum	419.2	410.8
Lunataspis aurora	455	445
Euproops anthrax	303.7	298.9
Limulus polyphemus	10	0
Paleolimulus signatus	303.7	279.3
Bembicosoma pomphicus	438.5	433.4
Bunodes lunula	426.2	422.9
Limuloides limuloides	423	419.2
Pasternakevia podolica	427.4	423
Cyamocephalus loganensis	433.4	427.4
Pseudoniscus roosevelti	427.4	419.2
Chasmataspis laurencii	468.1	460.9
Octoberaspis ushakovi	419.2	410.8
Stoermeropterus conicus	443.4	433.4
Eurypterus tetragonophthalmus	433.4	419.2
Rhenopterus diensti	416	397.5
Parastylonurus ornatus	438.5	423
Centruroides vittatus	0	0
Palaeophonus nuncius	433.4	430.5
Mastigoproctus giganteus	0	0
Galeodes armeniacus	0	0

SUPPLEMENTARY REFERENCES

1 Liu, Y. *et al.* Computed tomography sheds new light on the affinities of the enigmatic euarthropod Jianshania furcatus from the early Cambrian Chengjiang biota. *BMC Evolutionary Biology* **20**, 62 (2020).

2 Liu, Y. *et al.* Three-dimensionally preserved minute larva of a great-appendage arthropod from the early Cambrian Chengjiang biota. *Proceedings of the National Academy of Sciences* **113**, 5542–5546 (2016).

3 Liu, Y., Ortega-Hernández, J., Zhai, D. & Hou, X. A reduced labrum in a Cambrian great-appendage Euarthropod. *Current Biology* **30**, 3057–3061 (2020).

4 Saleh, F. *et al.* Taphonomic bias in exceptionally preserved biotas. *Earth and Planetary Science Letters* **529**, 115873 (2020).

5 Aria, C. & Caron, J.-B. Mandibulate convergence in an armoured Cambrian stem chelicerate. *BMC evolutionary biology* **17**, 261 (2017).

6 Ortega-Hernández, J., Van Roy, P. & Lerosey-Aubril, R. A new aglaspidid euarthropod with a six-segmented trunk from the Lower Ordovician Fezouata Konservat-Lagerstätte, Morocco. *Geological Magazine* **153**, 524–536 (2016).

7 Lerosey-Aubril, R., Zhu, X. & Ortega-Hernández, J. The Vicissicaudata revisited–insights from a new aglaspidid arthropod with caudal appendages from the Furongian of China. *Scientific Reports* **7**, 11117 (2017).

8 Legg, D. A., Sutton, M. D. & Edgecombe, G. D. Arthropod fossil data increase congruence of morphological and molecular phylogenies. *Nature Communications* **4**, 2485 (2013).

9 Sutton, M. D., Briggs, D. E., Siveter, D. J., Siveter, D. J. & Orr, P. J. The arthropod *Offacolus kingi* (Chelicerata) from the Silurian of Herefordshire, England: computer based morphological reconstructions and phylogenetic affinities. *Proceedings of the Royal Society of London. Series B: Biological Sciences* **269**, 1195–1203 (2002).

10 Haug, C. & Rötzer, M. A. The ontogeny of *Limulus polyphemus* (Xiphosura s. str., Euchelicerata) revised: looking "under the skin". *Development genes and evolution* **228**, 49–61 (2018).

11 Moore, R. A., Briggs, D. E. & Bartels, C. A new specimen ofWeinbergina opitzi (Chelicerata: Xiphosura) from the Lower Devonian Hunsriick Slate, Germany. *Paläontologische Zeitschrift* **79**, 399-408 (2005).

12 Stürmer, W. & Bergström, J. *Weinbergina*, a xiphosuran arthropod from the Devonian Hunsrück Slate. *Paläontologische Zeitschrift* **55**, 237–255 (1981).

13 Richter, R. & Richter, E. *Weinbergina opitzi* ng, n. sp., ein Schwertträger (Merost. Xiphos.) aus dem Devon (Rheinland). *Senckenbergiana* **11**, 193–209 (1929).

14 Selden, P. A., Lamsdell, J. C. & Qi, L. An unusual euchelicerate linking horseshoe crabs and eurypterids, from the Lower Devonian (Lochkovian) of Yunnan, China. *Zoologica Scripta* **44**, 645–652 (2015).

15 Selden, P. A. & Drygant, D. M. A new Silurian xiphosuran from Podolia, Ukraine, USSR. (1987).

16 Currie, L. On *Cyamocephalus*, a new synxiphosuran from the Upper Silurian of Lesmahago, Lanarkshire. *Geological Magazine* **64**, 153–157 (1927).

17 Anderson, L. I. A new specimen of the Silurian synziphosurine arthropod *Cyamocephalus*. *Proceedings of the Geologists Association* **110**, 211–216 (1999).

18 von Eichwald, E. *Die Grauwackenschichten von Liev-und Esthland*. (1854).

19 Krzemiński, W., Krzemińska, E. & Wojciechowski, D. Silurian synziphosurine horseshoe crab *Pasternakevia* revisited. *Acta Palaeontologica Polonica* **55**, 133–139 (2009).

20 Fortey, R. A. & Rushton, A. W. A new aglaspidid arthropod from the Lower Ordovician of Wales. *Palaeontology* **46**, 1031–1038 (2003).

21 Lerosey-Aubril, R., Ortega-Hernández, J., Kier, C. & Bonino, E. Occurrence of the Ordovician-type aglaspidid *Tremaglaspis* in the Cambrian weeks formation (Utah, USA). *Geological Magazine* **150**, 945–951 (2013).
22 Van Roy, P. *Non-trilobite arthropods from the Ordovician of Morocco*. PhD thesis, Ghent University (2006).
23 Van Roy, P., Briggs, D. E. & Gaines, R. R. The Fezouata fossils of Morocco; an extraordinary record of marine life in the Early Ordovician. *Journal of the Geological Society* **172**, 541–549 (2015).
24 Van Roy, P. *et al.* Ordovician faunas of Burgess Shale type. *Nature* **465**, 215–218 (2010).
25 Jago, J.B., García-Bellido, D.C. and Gehling, J.G. An early C ambrian chelicerate from the E mu B ay S hale, S outh A ustralia. *Palaeontology* **59**, 549–562 (2016).
26 Haug, J.T., Maas, A., Haug, C. and Waloszek, D. *Sarotrocercus oblitus*–small arthropod with great impact on the understanding of arthropod evolution. *Bulletin of Geosciences* **86**, 725–736 (2011).
27 Aria, C. & Caron, J.-B. A middle Cambrian arthropod with chelicerae and proto-book gills. *Nature* **573** 586–589 (2019).
28 Lamsdell, J. C. Revised systematics of Palaeozoic 'horseshoe crabs' and the myth of monophyletic Xiphosura. *Zoological Journal of the Linnean Society* **167**, 1–27 (2013).
29 Fusco, G., and Minelli, A., G. Arthropod Segmentation and Tagmosis. 9, 197-221 (2016). In Minelli, A., Boxshall, G. and Fusco, G. *Arthropod biology and evolution*. Springer-Verlag Berlin An (2016).
30 Dunlop, J. A. & Lamsdell, J. C. Segmentation and tagmosis in Chelicerata. *Arthropod structure & development* **46**, 395–418 (2017).
31 Selden, P.A., Lamsdell, J.C. and Qi, L. An unusual euchelicerate linking horseshoe crabs and eurypterids, from the Lower Devonian (Lochkovian) of Yunnan, China. *Zoologica Scripta* **44**, 645–652 (2015).
